# Supplementary material for: Automated microarray platform for single‐cell sorting and collection of lymphocytes following HIV reactivation
Source: Bioeng Transl Med. 2023 Jun 21;8(5):e10551. doi: 10.1002/btm2.10551 (PMC10487311; doi:10.1002/btm2.10551)
Supplement: Supplementary file 1 — Figure S1: Lymphocytes viability after 24 h without LRA (control, black) and under 1 μM of prostratin (orange), iBET151 (blue), and SAHA (magenta). LRA: prostratin (p > 1.0, 10 arrays, 8768 single cells), SAHA (p > 0.8, 4 arrays, 6354 cells), and iBET151 (p > 0.1, 4 arrays, 5118 cells). Figure S2: Variability and clustering of the mCherry intensity of reactivated HIV latency cells under prostratin. (a) mCherry intensity of each cluster over time. (b) Heat map plots showing the mean intensity values of all control, nonactivated, fast, and slow cells. Figure S3: Venn diagram showing all differentially expressed genes (DEGs) when applying a p‐value (<0.05) and log2 fold change (0.5) between reactivated versus non‐reactivated cells, as well as fast, slow versus control. [file BTM2-8-e10551-s001.pdf]

Supplementary information

Automated microarray platform for single-cell sorting and collection of lymphocytes following HIV reactivation

Belén Cortés-Llanos[1,2], Vaibhav Jain[3], Alicia Cooper-Volkheimer[2], Edward P. Browne[4,5,6], David M. Murdoch[2*], Nancy L. Allbritton[1*]

[1]Department of Bioengineering, University of Washington, Seattle, Washington, USA
[2]Department of Medicine, Duke University, Durham, North Carolina, USA
[3]Department of Molecular Physiology, Duke University, North Carolina, USA
[4]Department of Medicine, University of North Carolina, Chapel Hill, North Carolina, USA
[5]Department of Microbiology and Immunology, University of North Carolina, Chapel Hill, North Carolina, USA
[6]UNC HIV Cure Center, University of North Carolina, Chapel Hill, North Carolina, USA
*D. M. Murdoch: david.murdoch@duke.edu and N. L. Allbritton: nlallbr@uw.edu

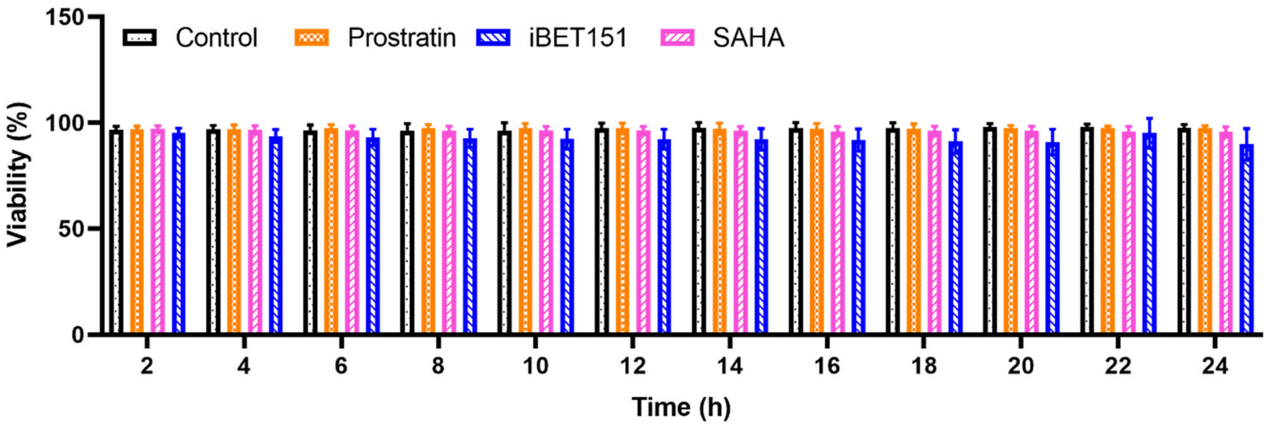

FIGURE S1: Lymphocytes viability after 24 hours without LRA (control, black) and under 1 µM of prostratin (orange), iBET151 (blue), and SAHA (magenta). LRA: prostratin (p>1.0, 10 arrays, 8768 single cells), SAHA (p>0.8, 4 arrays, 6354 cells), and iBET151 (p>0.1, 4 arrays, 5118 cells).

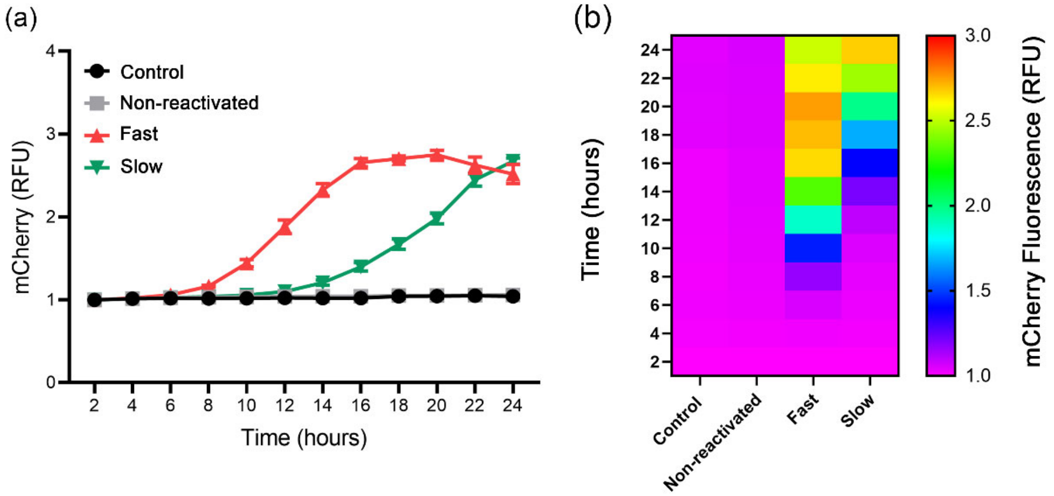

FIGURE S2: Variability and clustering of the mCherry intensity of reactivated HIV latency cells under prostratin. (a) mCherry intensity of each cluster over time. (b) Heat map plots showing the mean intensity values of all control, non-activated, fast, and slow cells.

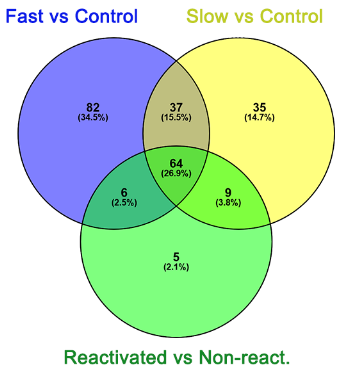

FIGURE S3: Venn diagram showing all differentially expressed genes (DEGs) when applying a p-value (<0.05) and log2 fold change (0.5) between reactivated vs. non-reactivated cells, as well as fast, slow vs. control.
